# Case Report: Atypical ocular surface squamous cell carcinoma

**DOI:** 10.3389/fmed.2026.1798013

**Published:** 2026-03-23

**Authors:** Tian Tian, Luyi Ying, Yuanfang Zhu, Wenya Qiu

**Affiliations:** Department of Ophthalmology, Sir Run Run Shaw Hospital, Zhejiang University School of Medicine, Hangzhou, Zhejiang, China

**Keywords:** atypical, misdiagnose, ocular surface, ocular surface squamous neoplasia, squamous cell carcinoma

## Abstract

Ocular surface squamous neoplasia (OSSN) is the most common ocular surface neoplasm. Ocular surface squamous cell carcinoma (OS-SCC) represents the invasive malignant component within the spectrum of OSSN. The patient in this case report presented with manifestations resembling herpes simplex keratitis at the initial visit, without the typical clinical features of OS-SCC. The diagnosis was revised after close follow-up, and the patient achieved improvement with anti-metabolite therapy. At the time of recurrence, the clinical manifestations were similar to scleral abscess, still without the typical features of OS-SCC. A definitive diagnosis was finally confirmed by surgery following close follow-up. This patient lacked typical clinical manifestations of OS-SCC in both episodes of onset. The diagnosis was revised based on close follow-up and a pathological biopsy, and the treatment regimen was adjusted in a timely manner, resulting in a favorable final prognosis.

## Introduction

Ocular surface squamous neoplasia (OSSN) represents the most prevalent ocular surface malignancy, with an annual incidence ranging from 0.03 to 1.9 cases per 100,000 individuals (1, 2). The primary risk factors associated with OSSN include exposure to ultraviolet B (UVB) radiation, human papillomavirus (HPV) infection, and human immunodeficiency virus (HIV) infection (3). These tumors pose a significant threat to both vision and life, as they have the potential to locally invade the cornea, sclera, uvea, eyelids, orbits, sinuses, and brain, as well as metastasize (4–6).

Ocular surface squamous cell carcinoma (OS-SCC) is the invasive malignant component of the OSSN spectrum. OS-SCC typically manifests as a papillary, leukoplakia, or gelatinous lesion involving the bulbar conjunctiva. Patients often present with non-specific symptoms such as redness, eye irritation, and visual disturbances when the visual axis is involved (3). The advancing margin of the tumor is usually light gray and avascular. When the cornea is involved, OS-SCC typically appears as a creamy-white epithelial lesion with irregular, scalloped borders. When against the white sclera, the pale gray avascular extension is nearly or completely invisible, and OS-SCC can present variably and has previously been misdiagnosed as refractory epithelial keratitis and scleral abscess (7, 8).

In this case, the patient did not exhibit the typical clinical manifestations of OS-SCC on either occasion. With close follow-up and surgical intervention, the diagnosis was refined, the treatment strategy was optimized, and a favorable prognosis was ultimately attained.

## Case report

**Figure 1:**
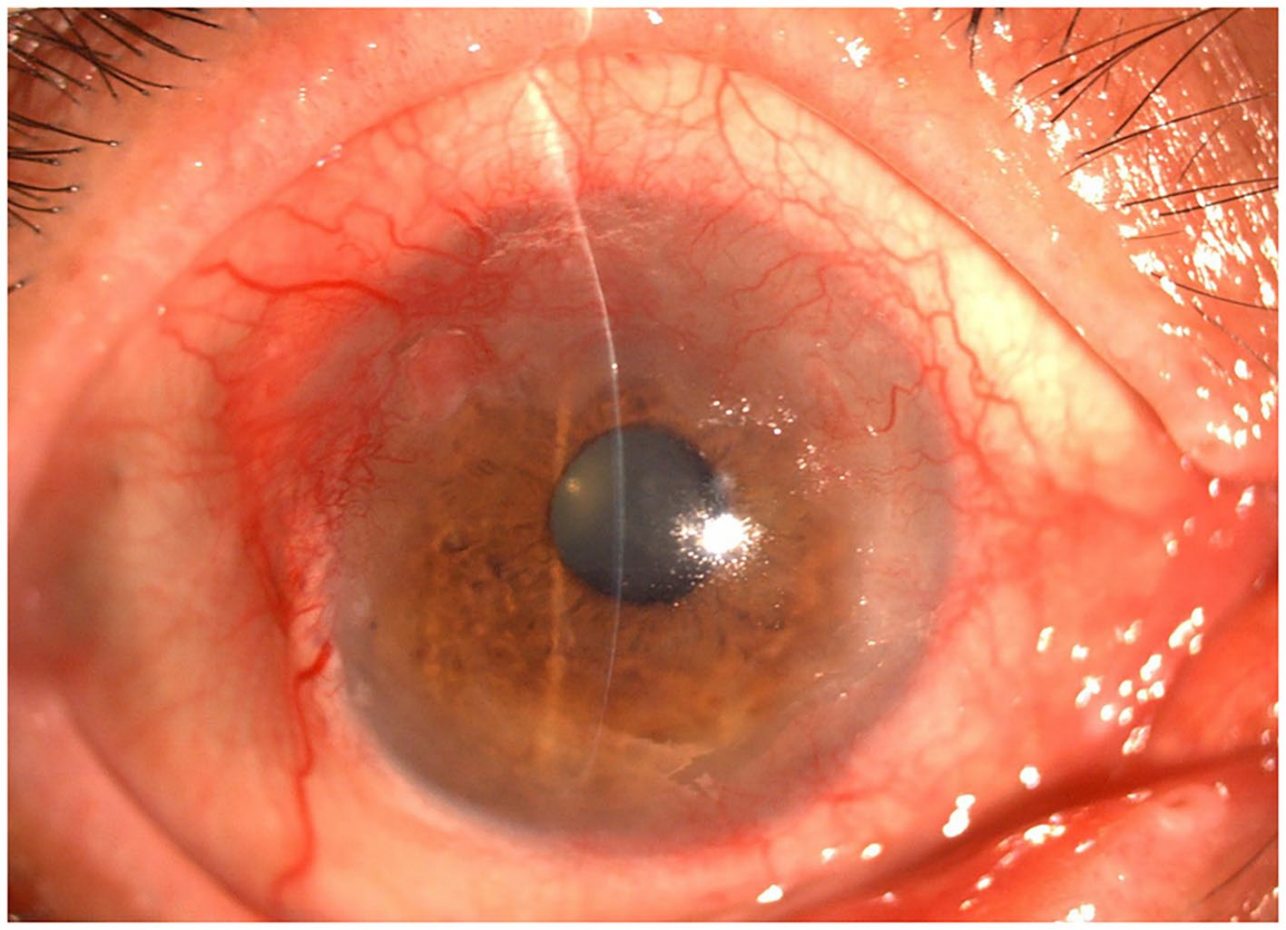
Anterior segment photographs at the initial onset. The lesion presented as an abnormally proliferative elevation extending along the limbus from the 8:30 to 6:30 clock positions, with a gelatinous surface and tortuous, dilated, abnormal vascular network. It invaded the cornea in a scalloped pattern, extending 2–4 mm inside the limbus; the superior portion of the lesion was wider but did not involve the pupillary area.

A 65-year-old man presented to the ophthalmology outpatient clinic with a 1-year history of recurrent foreign body sensation, photophobia, and blurred vision in the right eye, and self-reported a 2-year history of keratitis. The patient was a farmer with a history of pterygium resection in the right eye 4 years prior and hypertension for more than 10 years, and no other systemic medical history. The visual acuity of the right eye was counting fingers at 80 cm. Physical examination revealed marked conjunctival hyperemia, with limbal stromal opacity and stromal neovascularization. A geographical corneal epithelial defect was noted, and no obvious terminal bulb was observed. Based on the patient’s 2-year history of recurrent keratitis, an experienced corneal specialist initially diagnosed viral keratitis, and the patient was treated with oral acyclovir and 0.1% fluorometholone eye drops. Two weeks later, physical examination showed resolution of the epithelial defect, with residual punctate corneal epithelial defects. At 2-week follow-up, an obvious mass was observed on the corneal surface (Figure 1). The lesion presented as an abnormally proliferative elevation extending along the limbus from the 8:30 to 6:30 clock positions, with a gelatinous surface and tortuous, dilated, abnormal vascular network. It invaded the cornea in a scalloped pattern, extending 2–4 mm inside the limbus; the superior portion of the lesion was wider but did not involve the pupillary area. Based on the disease progression and the aforementioned morphological characteristics of the lesion, the attending physician suspected squamous cell carcinoma and initiated 0.02% mitomycin C (MMC) eyedrops four times daily according to the standard treatment guidelines (3). A treatment course consisted of a 1-week application followed by a 1-week break. After completing three treatment courses, the mass resolved, and the visual acuity was 0.5 (decimal).

**Figure 2:**
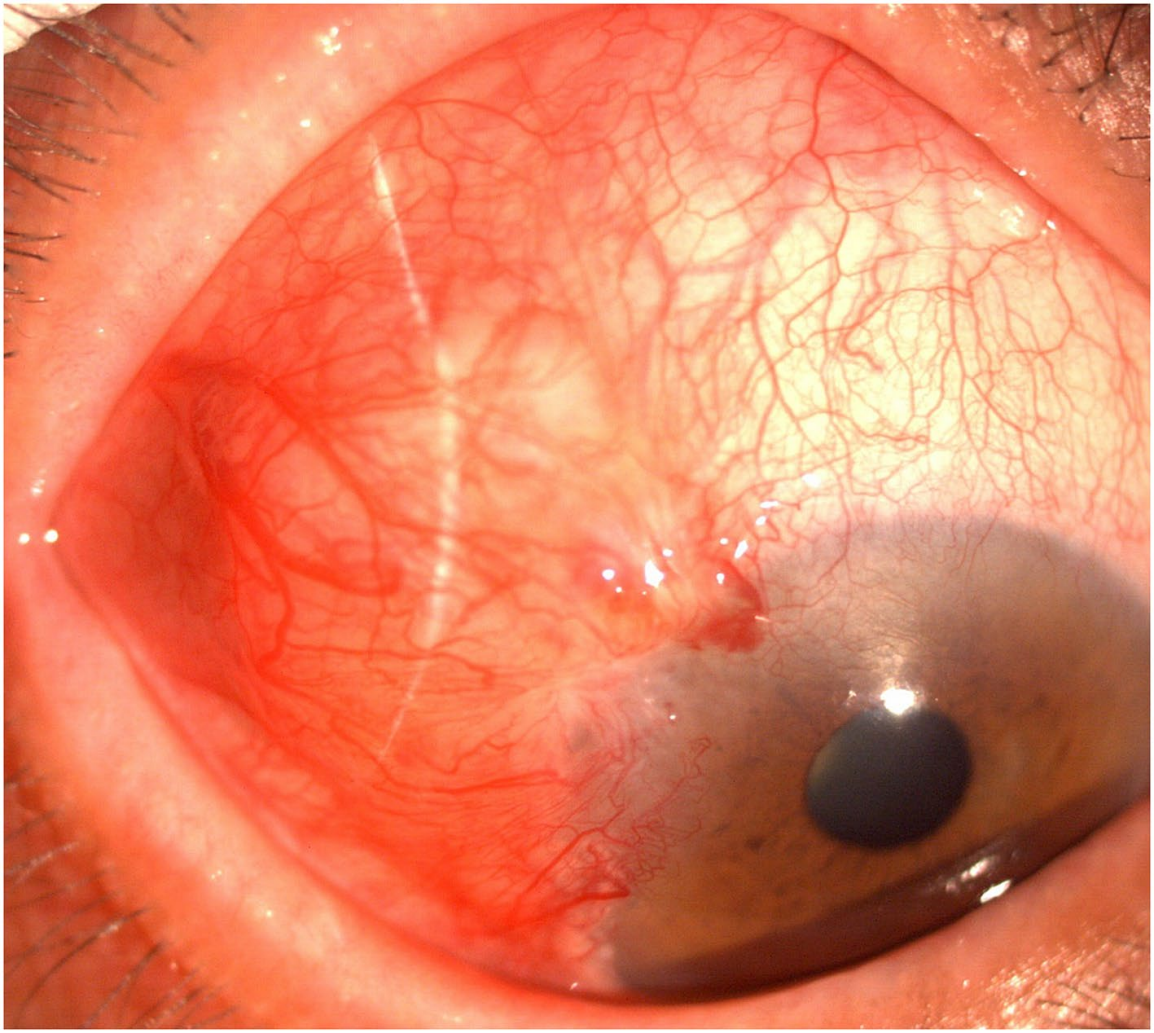
Anterior segment photographs at recurrence. The lesion manifested as a 6 × 7 mm localized elevation on the superotemporal sclera, involving approximately 1 mm inside the limbus; the lesion surface showed marked vascular engorgement and severe hyperemia.

**Figure 3:**
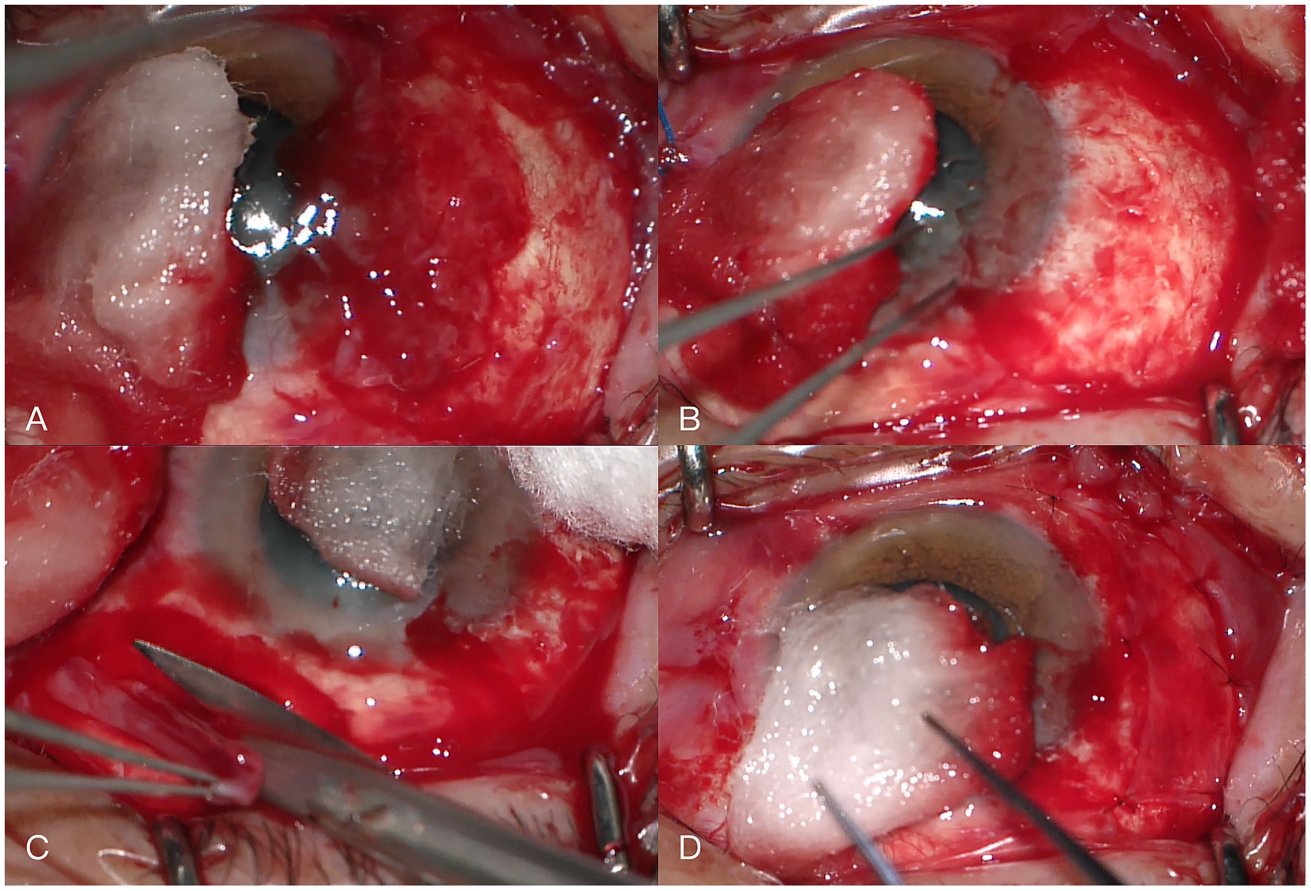
Intraoperative key images. **(A)** The lesion is located on the corneal-scleral surface with relatively clear boundaries and abundant superficial blood vessels. **(B)** The lesion was completely dissected from the corneal-scleral surface, and no intrascleral infiltration was observed. **(C)** A conjunctival graft measuring 2 × 6 mm was harvested from the superonasal bulbar conjunctiva. **(D)** The conjunctival graft was sutured *in situ* onto the bare temporal scleral surface.

**Figure 4:**
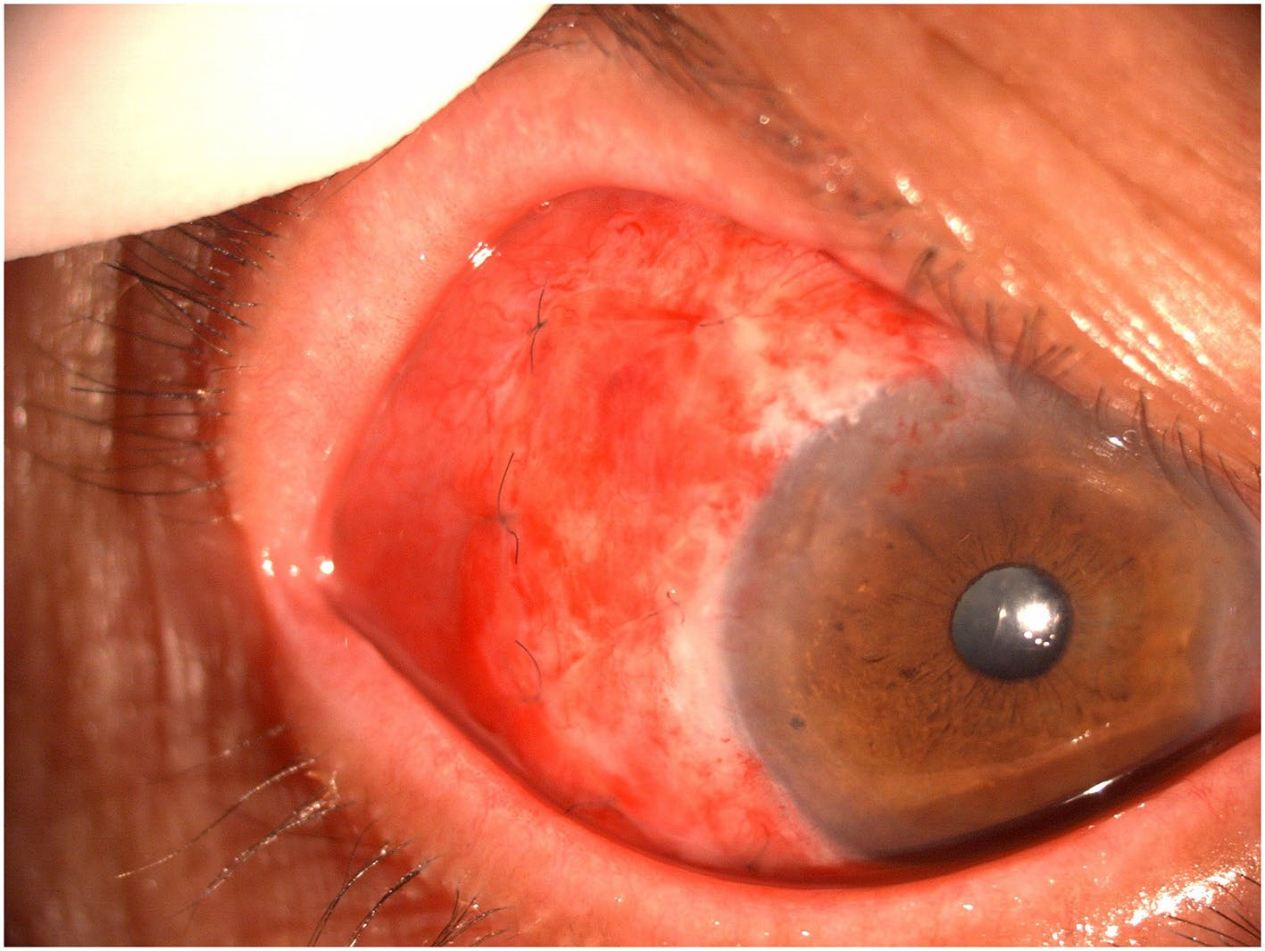
Clinical photograph of the ocular surface on the first postoperative day. A well-attached conjunctival graft with no residual ocular surface tumor is observed.

One year later, the patient returned to the same corneal disease expert clinic with a 1-month history of redness in the right eye (Figure 2). The lesion manifested as a 6 × 7 mm localized elevation on the superotemporal sclera, involving approximately 1 mm inside the limbus; the lesion surface showed marked vascular engorgement and severe hyperemia. The visual acuity of the right eye was 0.5 (decimal). The corneal epithelium appeared intact, smooth, and transparent. The diagnosis was considered suspicious for a scleral abscess. Treatment with 2% voriconazole eyedrops and an itraconazole tablet was administered. One week later, the mass showed no significant reduction in size, with the corneal epithelium remaining intact, smooth, and transparent, so the current treatment was continued. Two weeks later, the lesion had enlarged to approximately 7 × 8 mm, extending approximately 2 mm into the limbus, with a fish-flesh-like protrusion visible on its surface. Preoperative screening for HIV, syphilis, hepatitis C, and hepatitis B viruses was negative in the patient, with a normal immune status. Surgical intervention was performed, and the tumor was resected under local anesthesia. Intraoperatively, the lesion was confined to a localized area and could be completely dissected off the scleral surface with a clear demarcation from the sclera; no scleral invasion was identified. Therefore, simple excision was performed as the surgical approach. The no-touch technique was not adopted, nor was adjunctive cryotherapy administered. After tumor removal, a cotton sponge soaked in 0.02% MMC was applied to the affected corneal and scleral surfaces for 5 min, followed by thorough irrigation with copious normal saline. No secondary extended resection was conducted. A 2 × 6 mm conjunctival graft was harvested from the superonasal quadrant of the ipsilateral eye and transplanted onto the exposed superotemporal scleral surface (Figure 3). On postoperative day 1, the anterior segment photograph showed a well-attached conjunctival graft with no residual ocular surface tumor (Figure 4). Hematoxylin and eosin (H&E) staining and immunohistochemical analysis were performed on the surgically resected tumor tissue. The results demonstrated positive expression of CK-pan, P63, and P53, which provided crucial molecular evidence for the pathological diagnosis of OS-SCC (Supplementary Figure 1). The combined positive expression of these three markers was consistent with the characteristic morphological features of squamous cell carcinoma observed on H&E staining, forming a comprehensive pathological basis and confirming the definitive diagnosis of ocular surface squamous cell carcinoma in this case. According to the eighth edition of the American Joint Committee on Cancer (AJCC) Cancer Staging Manual, the patient’s OS-SCC was staged as T3 (9). Postoperatively, 0.02% mitomycin C (MMC) eye drops were applied four times daily (3). A treatment course consisted of 1-week application followed by 1-week break. After completing three treatment courses, it was stopped. At the 10-month follow-up, the right eye visual acuity was 0.6 (decimal), and the anterior segment photograph showed a smooth ocular surface without tumor recurrence (Supplementary Figure 2).

## Discussion

This case was initially diagnosed as herpes viral keratitis due to a history of recurrent episodes and an atypical geographic defect in the cornea. The epithelial defects were repaired after initial treatment, which was considered to rule out drug toxicity from previous multiple administrations. However, a subsequent mass gradually appeared, which was inconsistent with herpes viral keratitis, and was promptly diagnosed as a corneal mass and was treated with anti-tumor treatment. When the disease recurred, the clinical manifestations resembled a scleral abscess. However, the tumor did not subside after 3 weeks of treatment and gradually involved the cornea. The diagnosis was finally confirmed by post-surgical pathology. Immunohistochemical staining of the resected specimen showed positive expression of CK-pan, P63, and P53. These findings, together with the classic morphological characteristics identified on H&E staining, confirmed the diagnosis of OS-SCC, a type of OSSN. Previously reported cases of masquerading manifestations were typically characterized by lesions mimicking rapidly progressive necrotizing scleritis. Due to the strong invasiveness and deep involvement of the lesions, aggressive therapeutic approaches, such as enucleation or orbital exenteration, were generally required in clinical practice, making globe salvage difficult. Moreover, intervention was often delayed due to misdiagnosis, with pathological biopsy usually performed at the moderate to advanced stage of the disease (8, 10). In contrast, the case in this study lacked typical morphological features of OS-SCC, had a relatively indolent clinical course, and showed no deep scleral involvement or significant invasive manifestations. A definitive diagnosis was established via timely early surgical biopsy, followed by combined pharmacotherapy, which ultimately achieved successful globe salvage. This diagnostic and therapeutic process provides valuable clinical references for identifying and managing OS-SCC cases with indolent courses and atypical manifestations in clinical practice.

OS-SCC is traditionally diagnosed by biopsy, but some less invasive techniques can also help establish the diagnosis, such as high-resolution optical coherence tomography (HR-OCT), confocal microscopy, and specific vital staining. For instance, De Arrigunaga et al. (11) used HR-OCT to diagnose OS-SCC, which was verified by pathology. Liang et al. (12) identified OS-SCC through IVCM by the presence of dysplastic cells with hyper-reflective cytoplasm and a high nuclear to cytoplasmic ratio (13). Aliakbar Navahi et al. (14) reported that toluidine blue (TB) staining patterns have relatively high sensitivity and very high specificity in diagnosing OS-SCC. Steffen et al. (15) reported that methylene blue 1%, as a non-invasive *in vivo* stain, can serve as an auxiliary diagnostic tool to exclude malignant ocular surface lesions. For this patient, such non-invasive examinations might have helped achieve an earlier diagnosis, which is one of the limitations of this case report.

Surgical resection is conventionally regarded as the standard of care for OS-SCC. However, surgery can be associated with the development of complications such as limbal stem cell deficiency, symblepharon formation, conjunctival hyperemia, and conjunctival scarring (16–19). There is a burgeoning inclination toward medical management of OS-SCC, encompassing the application of topical chemotherapeutic agents and immunomodulatory agents, such as 5-fluorouracil (5-FU), MMC, and interferon alpha-2b (IFNα-2b). These pharmaceuticals can address the entire ocular surface and are used either alone or in conjunction with surgical procedures (20). The combination of surgical and medical modalities has demonstrated efficacy in OS-SCC cases at high risk of recurrence.

In this case, particularly when the patient returns for recurrence 1 year later, if we had used these non-invasive examinations, the right diagnosis may have been established earlier. This case underscores the tortuous path to accurate diagnosis and treatment for patients with atypical clinical presentations, highlighting the critical importance of close follow-up, timely correction of diagnosis, and prompt surgical intervention in the management of such conditions.

## Data Availability

The raw data supporting the conclusions of this article will be made available by the authors, without undue reservation.

## Supplementary material

SUPPLEMENTARY FIGURE 1 Pathological examination of the tumor included HE staining and immunohistochemical analysis. **(A)** Hematoxylin and eosin staining. **(B)** Pan-cytokeratin (CK-pan) immunohistochemistry. **(C)** P53 immunohistochemistry. **(D)** P63 immunohistochemistry.

SUPPLEMENTARY FIGURE 2 Final lesion status and ocular conditions at 10-month follow-up. A smooth ocular surface without tumor recurrence was observed.
