# Supplementary figures and images for: Case Report: Atypical ocular surface squamous cell carcinoma
Source: Front Med (Lausanne). 2026 Mar 23;13:1798013. doi: 10.3389/fmed.2026.1798013 (PMC13050655; doi:10.3389/fmed.2026.1798013)

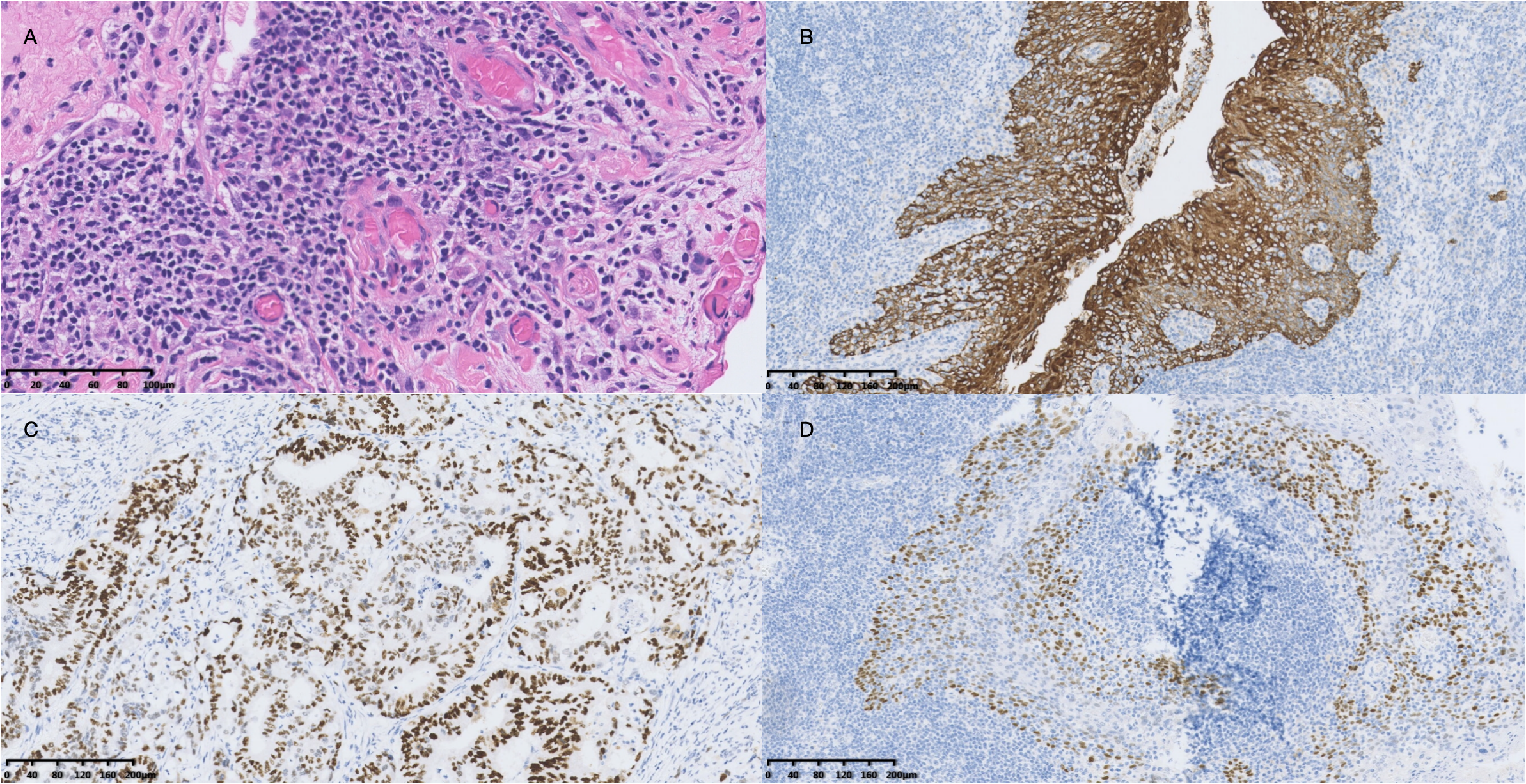

Supplement: SUPPLEMENTARY FIGURE 1 — Pathological examination of the tumor included HE staining and immunohistochemical analysis. (A) Hematoxylin and eosin staining. (B) Pan-cytokeratin (CK-pan) immunohistochemistry. (C) P53 immunohistochemistry. (D) P63 immunohistochemistry. [file Image_1.jpeg]

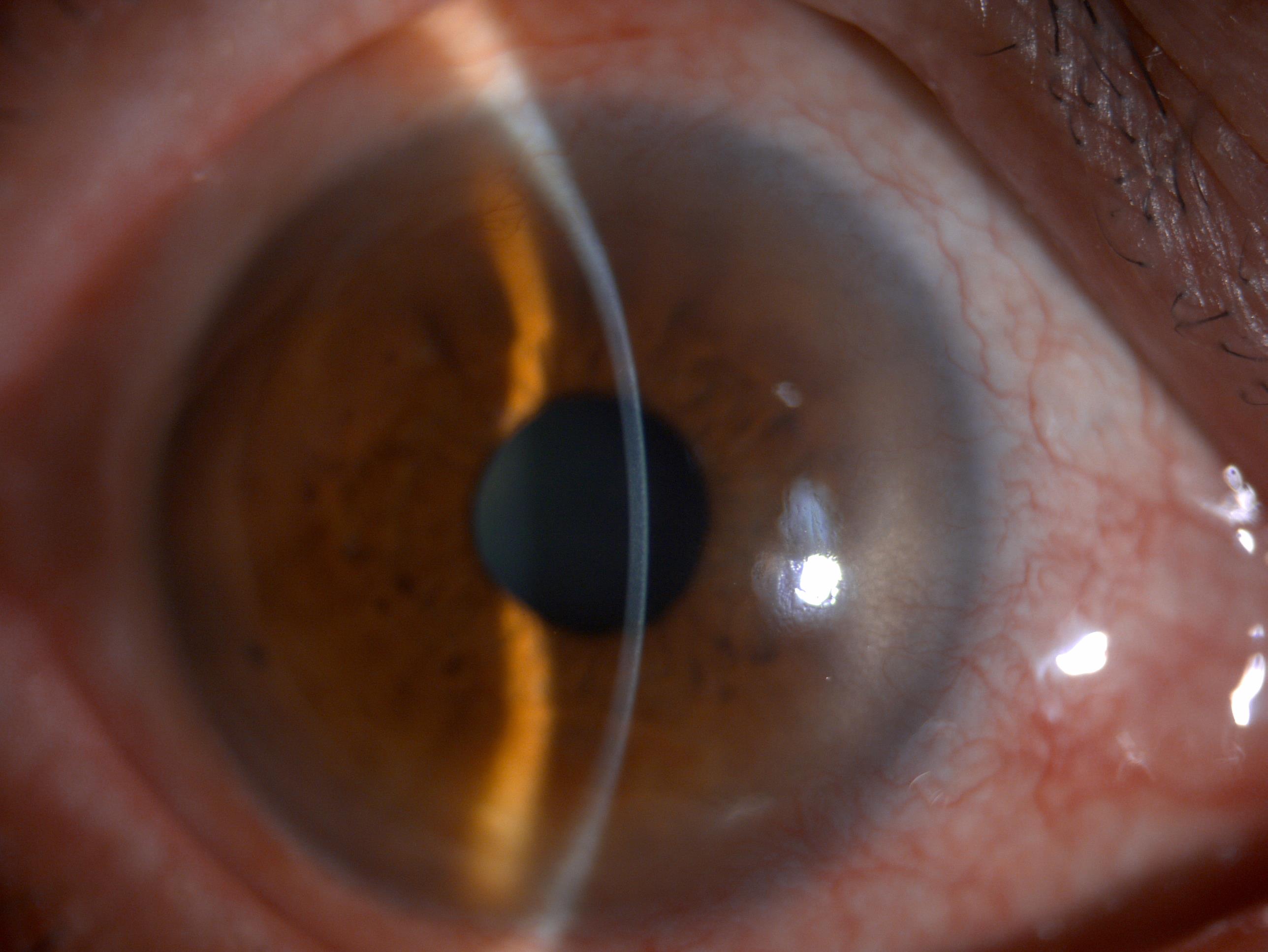

Supplement: SUPPLEMENTARY FIGURE 2 — Final lesion status and ocular conditions at 10-month follow-up. A smooth ocular surface without tumor recurrence was observed. [file Image_2.jpeg]
